# Using model-based geostatistics for assessing the elimination of trachoma

**DOI:** 10.1371/journal.pntd.0011476

**Published:** 2023-07-28

**Authors:** Misaki Sasanami, Benjamin Amoah, Adam Nouhou Diori, Abdou Amza, Abdoul Salam Youssoufou Souley, Ana Bakhtiari, Boubacar Kadri, Célia L. Szwarcwald, Daniela Vaz Ferreira Gomez, Ibrahim Almou, Maria de Fátima Costa Lopes, Michael P. Masika, Nassirou Beidou, Sarah Boyd, Emma M. Harding-Esch, Anthony W. Solomon, Emanuele Giorgi

**Affiliations:** 1 Lancaster Medical School, Lancaster University, Lancaster, United Kingdom; 2 Faculty of Medicine, School of Public Health, Imperial College London, London, United Kingdom; 3 Ophtalmologie de l’Hôpital Amirou Boubacar Diallo de Niamey, Niamey, Niger; 4 Faculty of Health Sciences, Abdou Moumouni University of Niamey, Niamey, Niger; 5 International Trachoma Initiative, Task Force for Global Health, Decatur, Georgia, United States of America; 6 Programme National de Sante Oculaire (PNSO), Niamey, Niger; 7 Institute of Scientific and Technological Communication and Information in Health, Oswaldo Cruz Foundation, Rio de Janeiro, Brazil; 8 Secretariat of Health and Environmental Surveillance, Ministry of Health, Brasília (DF), Brazil; 9 Ministry of Health, Lilongwe, Malawi; 10 National Blindness Prevention Program, Niamey, Niger; 11 Clinical Research Department, Faculty of Infectious and Tropical Diseases, London School of Hygiene & Tropical Medicine, London, United Kingdom; 12 Global Neglected Tropical Diseases Programme, World Health Organization, Geneva, Switzerland; Federal University of Ceará, Fortaleza, Brazil, BRAZIL

## Abstract

**Background:**

Trachoma is the commonest infectious cause of blindness worldwide. Efforts are being made to eliminate trachoma as a public health problem globally. However, as prevalence decreases, it becomes more challenging to precisely predict prevalence. We demonstrate how model-based geostatistics (MBG) can be used as a reliable, efficient, and widely applicable tool to assess the elimination status of trachoma.

**Methods:**

We analysed trachoma surveillance data from Brazil, Malawi, and Niger. We developed geostatistical Binomial models to predict trachomatous inflammation—follicular (TF) and trachomatous trichiasis (TT) prevalence. We proposed a general framework to incorporate age and gender in the geostatistical models, whilst accounting for residual spatial and non-spatial variation in prevalence through the use of random effects. We also used predictive probabilities generated by the geostatistical models to quantify the likelihood of having achieved the elimination target in each evaluation unit (EU).

**Results:**

TF and TT prevalence varied considerably by country, with Brazil showing the lowest prevalence and Niger the highest. Brazil and Malawi are highly likely to have met the elimination criteria for TF in each EU, but, for some EUs, there was high uncertainty in relation to the elimination of TT according to the model alone. In Niger, the predicted prevalence varied significantly across EUs, with the probability of having achieved the elimination target ranging from values close to 0% to 100%, for both TF and TT.

**Conclusions:**

We demonstrated the wide applicability of MBG for trachoma programmes, using data from different epidemiological settings. Unlike the standard trachoma prevalence survey approach, MBG provides a more statistically rigorous way of quantifying uncertainty around the achievement of elimination prevalence targets, through the use of spatial correlation. In addition to the analysis of existing survey data, MBG also provides an approach to identify areas in which more sampling effort is needed to improve EU classification. We advocate MBG as the new standard method for analysing trachoma survey outputs.

## Author summary

Trachoma is the most common infectious cause of blindness worldwide. Achieving elimination in resource-limited settings requires pragmatic strategies, including determining the likelihood that the elimination prevalence targets have been reached. Model-based geostatistics (MBG) is a branch of spatial statistics that can underpin highly efficient methods for designing surveys and analysing surveillance data for NTD programmes. Here, we illustrate the application of the MBG framework to analyse trachoma surveillance data from Brazil, Malawi, and Niger. Using the elimination criteria set by WHO, we predict the likelihood of elimination thresholds for trachomatous inflammation—follicular (TF) and trachomatous trichiasis (TT) having been achieved in each evaluation unit in each of the three countries. MBG is a statistically rigorous approach to quantify the likelihood around the exceedance of elimination prevalence thresholds. By providing a way to identify areas where there is more uncertainty about the achievement of elimination, MBG could be used to select areas in which more intensive sampling efforts should be undertaken.

## Introduction

Trachoma, one of twenty neglected tropical diseases (NTDs), remains the leading infectious cause of blindness globally [1,2]. It is caused by the bacterium *Chlamydia trachomatis*, which is transmitted from person to person by ocular and nasal secretions of infected people, during direct contact between individuals, or indirectly via flies or fomites [3–5]. Trachoma is prevalent in the most deprived and marginalised communities where crowded living conditions are common and access to clean water and sanitation is limited [2,6–9]. In endemic areas, trachomatous inflammation—follicular (TF), a sign of active trachoma, is common among children aged 1–9 years [2,10]. After years of repeated infections, some individuals develop prominent conjunctival scarring and have their upper eyelids turn inward so that the eyelashes rub against the globe. This is referred to as trachomatous trichiasis (TT), which can require surgery to prevent visual impairment and blindness [2]. TT and loss of vision are generally more common in women than in men [11,12], since the former are more likely to care for young children and therefore be exposed to more episodes of infection [2]. Although blindness caused by trachoma is generally considered irreversible, it is possible to prevent it, primarily using the surgery, antibiotics, facial cleanliness, and environmental improvement (SAFE) strategy promoted by the World Health Organization (WHO) [13,14].

In 1996, WHO launched the WHO Alliance for the Global Elimination of Trachoma by 2020 (GET2020) to eliminate trachoma as a public health problem [15]. Elimination is defined as: (i) a prevalence of TT unknown to the health system < 0.002 (0.2%) in adults aged ≥ 15 years, in each formerly endemic evaluation unit (EU) and (ii) a prevalence of TF < 0.05 (5%) in children aged 1–9 years, in each formerly endemic EU; plus (iii) the presence of a system to identify and manage incident cases of TT, which are expected to arise for many years after the prevalence thresholds (i) and (ii) are met. An EU for assessment of elimination is defined as the administrative unit for health care management, which typically contains a population of between 100,000 and 250,000 persons [16]. In the standard approach, the age- or age- and gender-specific prevalences of TF among children aged 1–9 years or TT among adults aged ≥ 15 years, respectively, are calculated. They are then standardised using the proportion of population expected to have that age or age-gender, according to the most recent census data available [17]. At the time of acceptance of this paper in June 2023, 17 countries had been validated by WHO as having eliminated trachoma as a public health problem [18].

The decision on whether a country has achieved the elimination criteria has to be informed by accurate and precise estimates of disease prevalence. This can be challenging in settings with very low prevalence and spatially sparse data, which is often the case in trachoma surveys. A possible solution to this is to sample a larger proportion of the population, however, this is usually infeasible due to resource constraints. The 4^th^ Global Scientific Meeting on Trachoma recommended that national programmes could combine data from multiple adjacent EUs [19], which would allow improvement in estimates of disease prevalence at one location by (for example) using information from nearby EUs. Model-based geostatistics (MBG) [20] is an established set of spatial statistical methods that has been increasingly used in low-resource settings to inform disease control programmes. In a recent study of TT mapping in Ethiopia, MBG methods were used to assess the elimination status of EUs; it was shown that this approach yielded substantially more precise estimates of TT prevalence compared to the standard trachoma prevalence survey approach [21].

The objective of this paper is to demonstrate the general applicability of MBG methods to assess the elimination of trachoma in different settings. To this end, we predict TF and TT prevalence using trachoma data collected from Brazil, Malawi, and Niger. These countries were selected as they differ in their trachoma elimination status, as described in detail in the Methods section below. Through these three case studies, we demonstrate how statistically rigorous MBG methods are used for borrowing the strength of information across space and making the best possible use of spatially sparse trachoma survey data. We also provide a framework to guide the inclusion of gender and age effects in MBG for trachoma.

## Methods

### Country settings

Brazil: From the 18^th^ to the early 20^th^ century, trachoma was spread through migrant populations in Brazil’s Northeast and the São Francisco Valley. The Federal Government led control campaigns from 1923 to 1998 [22], and in the 1970s, trachoma was considered eradicated in São Paulo. This belief (in eradication) became generalised across the entire country, leading to decreased engagement in surveillance and control activities. To better understand the contemporary burden of trachoma among children, school surveys were implemented in municipalities with Human Development Index (HDI) below the national mean in the early 2000s [23–25]. A nationwide study [26] found that 11 (41%) of 27 surveyed states had a prevalence of TF ≥ 5% (although participants included those aged ≥ 10 years). These results led to strengthening of national surveillance and control activities for trachoma [26,27], including antibiotic treatment of individuals with active trachoma and their contacts, identified through active case finding and contact tracing. This contributed to a marked decline in the TF prevalence between 2008 and 2016, as evidenced by the Brazil Information System for Notifiable Diseases (SINAN) [27]. A recent study showed that the prevalences of TF and TT were below the target for elimination in eight of nine surveyed non-indigenous EUs in 2018–19 [27]. In this most recent survey series, as is traditional, prevalence was estimated using a standard statistical approach that adjusts for age for TF, and age and gender for TT [17]. The prevalence among indigenous communities has not been recently estimated; this is currently under investigation.

Malawi: Since the 1980s, Malawi has recognised trachoma as an endemic disease [28,29]. A population-based survey conducted in two districts in Central and Southern Malawi in 2008 showed a prevalence of TF ≥ 10% (a threshold prevalence defined by WHO for determining the duration of annual mass drug administration [MDA]) and TT ≥ 0.2%, indicating trachoma was a public health problem [30]. Based on these findings, the Ministry of Health launched its first national-level trachoma control programme to implement the SAFE strategy, whilst stressing the need to estimate prevalence in other regions. Through surveys conducted between 2013 and 2015 with the support of the Global Trachoma Mapping Project (GTMP), trachoma mapping was officially completed in all suspected endemic areas, showing that some EUs in Central and Southern Malawi exceeded the TF and TT elimination thresholds [31,32]. Subsequently, the country intensified its efforts to eliminate trachoma and in 2022 was validated by WHO as having eliminated the disease, based on TF and TT prevalences [33].

Niger: After identifying almost all regions as being endemic for trachoma, Niger started SAFE implementation in 1999 and expanded it nationally in 2009 [34]. In order to determine eligibility for district- or sub-district-wide SAFE implementation, 31 district-level trachoma prevalence surveys were conducted from 2009 to 2012. The prevalence of TT in ≥ 15-year-olds ranged from 0.1–5.4% and the prevalence of TF in 1–9-year-olds ranged from 0.1–42.4%, suggesting the need for continued SAFE interventions in 16 districts, primarily in eastern Niger [34]. As of June 2022, 41 out of 72 health districts were identified as having TT prevalence ≥ 0.2%, and a combined population of more than three million people required the A, F and E interventions to reduce EU-level TF prevalence to < 5% [35].

### Data

We obtained the data from trachoma baseline, impact and pre-validation surveillance surveys [36,37] conducted in Brazil, Malawi, and Niger. All surveys were supported by Tropical Data and used a standardised two-stage cluster sampling methodology as defined by the GTMP [17,36,38]. Briefly, the first stage involves selection of 20–30 clusters (villages) using a probability-proportional-to-size sampling method, followed by the second-stage selection of approximately 30 households within each cluster, using compact segment, systematic, or random sampling. Consenting residents had both eyes examined for TF and TT using WHO’s simplified grading system for trachoma [39]. A case of TT was defined as an individual aged ≥ 15 years who had at least one eyelash touching the eyeball or showed evidence of recent epilation of in-turned eyelashes. Cases were excluded if the individuals (i) had TT post-operatively, (ii) had refused surgery, or (iii) were listed for surgery but had not yet received an operation.

Specifically, we used the data from nine Brazil EUs surveyed in 2018–19, 18 Malawi EUs surveyed in 2017–19, and 85 Niger EUs surveyed in 2017–19. The Brazil data were exclusively from baseline (i.e. pre-intervention) surveys. In Malawi and Niger, depending on the EU, impact and/or surveillance (i.e. post-intervention) surveys were available. When both impact and surveillance surveys were available in the same administrative unit, we just used the data from the most recent survey. As a result of this, only surveillance surveys were analysed in the case of Malawi. In the analysis for Brazil, we focused on the six EUs of north-eastern Brazil, namely Nordeste Paraense, Leste Maranhense, Noroeste Cearense, Sertão Pernambucano, Sertão Alagoano, and Vale São do Francisco da Bahia. We did not include the data from the north part of Brazil, from Vale do Jurua, Sudoeste Amazonense, and Norte de Roraima, because in those EUs, only 13 TF cases were detected in 2,318 examined children, and only one TT case was detected in 5,891 examined individuals aged ≥ 15 years, making the use of any statistical model infeasible.

### Geostatistical model

We developed geostatistical Binomial models for prevalence of TF and TT that account for age (for TF) or age and gender (for TT), as fixed effects, and for unexplained Binomial extra variation through the use of random effects. We express the general form of the models for TF and TT as follows.


logoddsofTFforanindividual=effectsofage+spatiallycorrelatedresidualvariation+spatiallyuncorrelatedresidualvariation

logoddsofTTforanindividual=effectsofage+effectsofgender+spatiallycorrelatedresidualvariation+spatiallyuncorrelatedresidualvariation

Based on existing scientific evidence and also guided by a preliminary exploratory analysis, we have summarised our approach for the introduction of age and gender effects in Table 1. In the model for TF, we defined the trend of age effect using a linear spline with a knot at the age of 3 years, since it is expected that prevalence should increase until around age 2–4 years followed by a decline [2,10]. This expectation was supported by our data on TF for Malawi and Niger, with the highest prevalence at age 3 years. In Brazil, prevalence showed a steady increase with increasing age, hence, we introduced age as a logit linear effect. The reason for preferring linear splines over other smoothing techniques is because of their greater interpretability for the effects of the covariates on trachoma prevalence [40]. Also, in the models for TF, we did not introduce any gender effect, since there is no strong scientific evidence to support a consistent difference in exposure to *C*. *trachomatis* between male and female children [2].

**Table 1:** Effects of age and gender on trachomatous inflammation—follicular (TF) and trachomatous trichiasis (TT) prevalence on logit scale.

Variables	TF	TT
Age	Included as a linearly increasing trend until around age 2–4 years, followed by a linearly decreasing trend. If this was not supported by data, assumed a linearly increasing trend	Included as a linearly increasing trend
Gender	Not included	Included
Age-gender interaction	Not included	Included if statistically significant

In the model for TT, we controlled for gender [11,12,41] regardless of statistical significance level, and introduced age as logit linear effect [2,10]. However, we did not introduce the gender effect in Brazil because it was not possible to distinguish between prevalence in males and females due to the small number of cases detected. Because differences in age trends could be observed between males and females for TT due to differences in mean exposure to *C*. *trachomatis* [41–43] and possibly sex-related biological phenomena [44], we decided to include an age-gender interaction in the model if this was statistically significant at the 95% confidence level. The final form of the model for each sign and country is described in Table A in S1 Text.

All data analysed in this study showed evidence of residual spatial correlation for both TF and TT. To address this, the geostatistical models fitted to the data included two types of random effects. More specifically, we included a spatial random effect, modelled as a Gaussian process, and unstructured random effects, modelled as Gaussian noise. The former accounts for between-cluster variation whilst the latter accounts for within-cluster variation.

We predicted local TF and TT prevalence by first laying grid squares over the EUs and computing prevalence for each age or age-gender class within each grid. We defined the areas shown in Fig A in S1 Text as EUs for prevalence prediction in this study, and the spatial resolution was determined based on the estimated scale of the spatial correlation for each country (see S1 Text). To allow for comparison with the standard approach [17], we then standardised prevalence for age bands of one-year for TF, and gender-specific five-year age bands for TT, using EU-specific population census data in Brazil (2010 census) [45] and national census data in Malawi (2018 census) and Niger (2012 census) [46,47]. To account for spatial heterogeneities in population density in generating the EU-wide standardised average prevalence for TF and TT, we weighted the predictions for each pixel using the population density data obtained from WorldPop [48].

Finally, we obtained 10,000 predictive samples for the EU-wide standardised average prevalence, for both TF and TT, and used the 10,000 samples to compute: the point prediction of the EU-wide standardised average prevalence, using the mean of the predictive samples; the 95% confidence-level prediction intervals; and the probability of elimination having been achieved, computed as the proportion of predictive prevalence samples that fell below the elimination threshold. We used R for all analyses, including the package “PrevMap” [49] to perform the geostatistical analysis. Technical details of the approach are provided in S1 Text.

## Results

Table 2 shows crude (unadjusted) TF and TT prevalence in Brazil, Malawi, and Niger. For both TF and TT, Brazil had the lowest crude prevalence whilst Niger had the highest. The crude TF prevalence by country, including data from all available EUs in that country, ranged from 0.44% to 3.66%. For TT, the prevalence was higher among females than males in Malawi and Niger, although this gender difference was not observed in Brazil, where only 11 cases were detected amongst 12,603 people aged ≥ 15 years examined.

**Table 2:** Crude prevalence of trachomatous inflammation—follicular (TF) and trachomatous trichiasis (TT) in Brazil, Malawi, and Niger.

Disease	Study population	Crude prevalence (%)		
		Brazil	Malawi	Niger
TF	Children aged 1–9 years	0.44% (16/3,666)	1.65% (301/18,283)	3.66% (5,369/146,790)
TT unknown to the health system	Males aged ≥ 15 years	0.12% (7/5,612)	0.06% (6/10,410)	0.16% (84/51,629)
	Females aged ≥ 15 years	0.06% (4/6,991)	0.19% (32/16,741)	0.41% (331/81,086)

**Fig 1:**
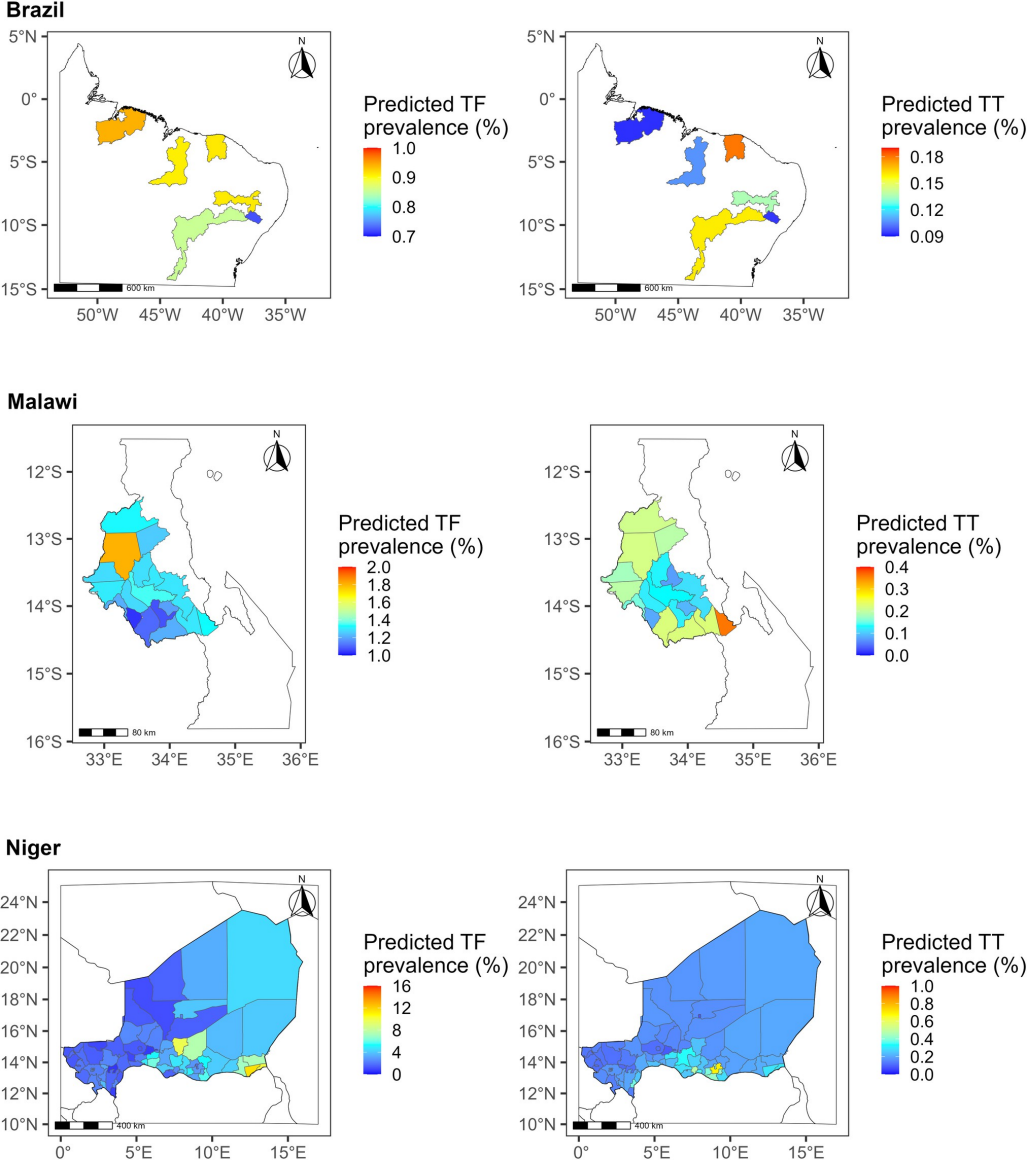
Predicted trachomatous inflammation—follicular (TF) and trachomatous trichiasis (TT) prevalence in Brazil, Malawi, and Niger. The boundaries and names shown and the designations used on this map are based on Global Administrative Areas (GADM) [50] and do not imply the expression of any opinion whatsoever on the part of the authors, or the institutions with which they are affiliated, concerning the legal status of any country, territory, city or area or of its authorities, or concerning the delimitation of its frontiers or boundaries.

According to the models, point predictions for both TF and TT prevalence in Brazil were below the elimination thresholds (Fig 1). In Malawi, the predicted TF prevalences were below the threshold, whereas six areas had a TT prevalence above the threshold. In Niger, the North-western regions had lower predicted TF prevalence and the South-eastern had higher, ranging from 0.5% to 15.7%. A similar trend was observed for TT prevalences.

**Fig 2:**
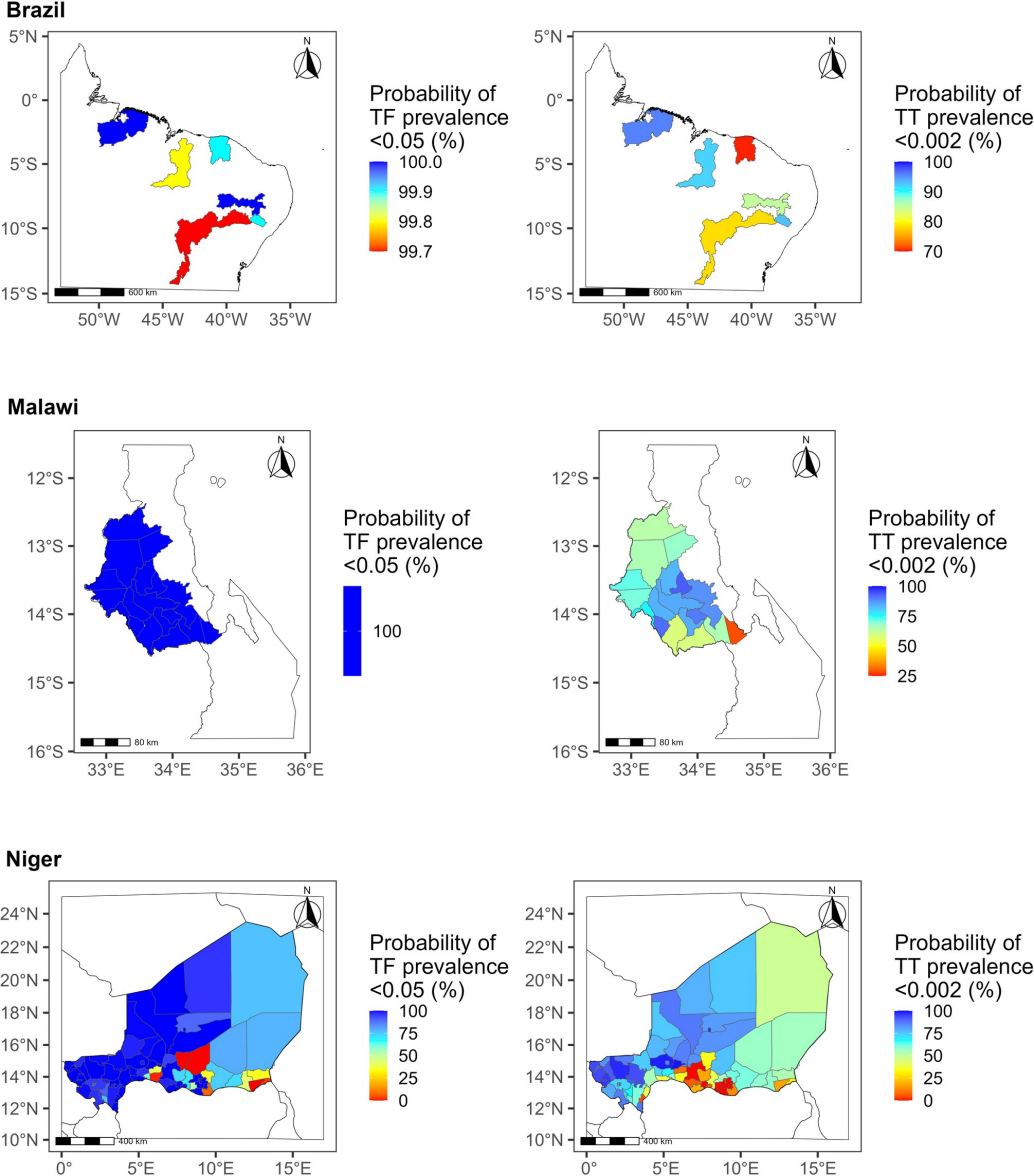
Probability of having achieved the elimination target for trachomatous inflammation—follicular (TF) and trachomatous trichiasis (TT) prevalence in Brazil, Malawi, and Niger. The boundaries and names shown and the designations used on this map are based on Global Administrative Areas (GADM) [50] and do not imply the expression of any opinion whatsoever on the part of the authors, or the institutions with which they are affiliated, concerning the legal status of any country, territory, city or area or of its authorities, or concerning the delimitation of its frontiers or boundaries.

We found that in Brazil, it is highly likely that TF prevalence met the target across all EUs (Fig 2). The probability of TT being below the elimination threshold (<0.2%) was more than 90% in three EUs out of six. For Sertão Pernambucano, Vale do São Francisco da Bahia, and Noroeste Cearense, the probability was 85%, 78%, and 70%, respectively. Similarly for Malawi, models indicated that the elimination criterion for TF had been met with greater than 95% likelihood. However, results are more uncertain for TT in one EU in particular, namely Dedza East, where the likelihood of achievement of elimination was 28%. In Niger, the probability for TF and TT spanned the full range of values, from ~0% to 100%. For TF, 65 of 107 EUs had likely met the elimination target with a probability of more than 95%, although 20 EUs likely had not, with a probability of having met the elimination target of less than 50%. For TT, 8 EUs had a more than 95% likelihood, whilst 38 EUs had less than 50%.

## Discussion

We have demonstrated the use of MBG methods for assessing trachoma elimination through three case studies, involving data from Brazil, Malawi, and Niger. Our approach can be adapted to different contexts to reflect data availability and age-gender trends, which can vary substantially across countries. We also showed that MBG methods can be applied to different epidemiological settings, ranging from very low prevalence settings such as Brazil and Malawi to relatively high prevalence settings such as Niger. One crucial aspect in which our MBG framework differs from the standard approach for the analysis of trachoma prevalence survey data is that it provides a probability statement on the exceedance or not of the elimination threshold. Quantification of uncertainty is essential to better inform the decision-making process and identify areas where the data do not provide enough information on achievement of elimination. This statistical aspect is ignored by the standard approach. For this reason, we argue that the model-based geostatistical approach presented in this paper is an important methodological improvement that answers more directly the problem of achievement of elimination for TF and TT in a given EU.

As noted at the 4^th^ Global Scientific Meeting on Trachoma, an efficient survey analysis strategy is vital for trachoma programmes [19]. As prevalence decreases, it becomes more challenging to precisely predict the prevalence without increasing the sample size. MBG provides one of the most efficient solutions to this, by allowing unsampled locations to borrow information from neighbouring sampled locations [51–53]. We show here that MBG can be effectively used in different epidemiological settings, both with very low trachoma prevalence, such as Brazil and Malawi, and relatively high prevalence, such as Niger. Future extensions of the proposed MBG framework will aim to incorporate spatially referenced covariate effects to account for well-established factors associated with trachoma, such as temperature, elevation, and precipitation [54]. However, whilst it is generally good practice to use covariates to aid spatial predictions of prevalence, it can be problematic in the context of low prevalence. This is because, as prevalence declines, the association between covariates and disease becomes more difficult to discern empirically and we would be wary of applying poorly estimated regression relationships to predict prevalence at unsampled locations. For example, in our case studies, the low levels of prevalence observed in Brazil and Malawi would likely make the use of covariates infeasible.

In the case of Brazil, the results obtained from our MBG approach are largely comparable to those generated using the standard approach (which have already been made publicly available [27]). Indeed, we observed that the 95% prediction intervals of the EU-wide standardised average prevalence from our MBG models overlap with those from the standard approach (see S1 Text), with the only exceptions being the prevalence estimates previously reported as 0% [27] and from Vale do São Francisco da Bahia, where there was only one TF case amongst 614 examined. Using the standard approach, we would conclude that prevalence was below the elimination threshold for both TF and TT in almost all EUs. However, our MBG approach, which also considers the uncertainty in the EU-wide prevalence predictions, indicated that although all EUs achieved TF prevalence < 5% with nearly 100% confidence, there was some uncertainty that TT prevalence was < 0.2%. The highest level of uncertainty was in Noroeste Cearense, where we found a likelihood of TT elimination of 70%. More data might be collected from this EU to establish without ambiguity whether the TT elimination prevalence threshold has been reached.

Malawi’s results from our analysis were mostly in accordance with WHO’s validation of it having achieved the elimination goal (for which the data from the most recent surveys were also used) [33]. The model indicated that the elimination criterion for TF was met with greater than 95% likelihood, as shown in Fig 2. For TT prevalence, however, the point predictions were above the elimination threshold of 0.2% for six EUs analysed, and it was shown that there was more uncertainty in the achievement of elimination particularly in Dedza East. The MBG analytical approach provides more tangible quantification of uncertainty in prevalence predictions, facilitating more informed discourse about exactly how much uncertainty policymakers are willing to tolerate, whilst also identifying the areas where sustained monitoring and resource allocation should be targeted even after elimination has been attained.

In all three countries, we found evidence of residual spatial correlation in the data which justified the use of MBG. However, in other settings, it is possible that the data will not show strong evidence of this, making the estimation of MBG models more difficult. In that scenario, a model-based approach for the estimation of EU-wide prevalence might still be achieved by simplifying the structure of our statistical models. More specifically, the spatial Gaussian process component which is introduced in all our models could be removed and extra-Binomial variation could be accounted for using unstructured random effects only. When using this simpler model, the generation of EU-wide standardised prevalence estimates and probabilities of elimination are obtained following the approach illustrated in this paper with only minimal technical adjustments.

When generating the EU-wide average prevalence using the illustrated model-based geostatistical approach, each pixel is weighted according to the WorldPop population density estimates. As a result of this, we should point out that the WorldPop data are based on model predictions and therefore could be uncertain if the input population data are not recent and/or not accurate due to considerable subnational variations in migration, fertility, and mortality [55].

As shown here, the MBG process must be guided by both contextual knowledge and empirical data exploration. These were especially important when deciding how to incorporate age and gender. For example, in our analysis for Brazil, TF prevalence showed a steady increase with increasing age, which differed from the pattern observed in other settings [30,41,56]. Hence, unlike for Malawi and Niger, we decided to include age as a logit linear effect which we justify as follows. First, the data were not informative enough to allow us to estimate a more complex relationship between prevalence and age. Second, individuals diagnosed as having TF might have conjunctivitis precipitated by a stimulus other than *C*. *trachomatis* infection. Scientific understanding of the diseases that cause a similar presentation to TF is currently incomplete [57–59], and disentangling them from trachoma requires further research. Finally, for the TT analysis in Brazil, we did not include gender effects. We based this decision on the fact that gender differences were so small that we could not precisely estimate them from the data. We also point out that the assumption of a logit linear trend in age for TT prevalence should be carefully assessed, especially in older ages. For example, in settings where TT surgery is offered to a population and awareness of, acceptance of and access to the service is high, age effects might be obscured.

MBG has been used for the analysis of various infectious diseases [60–67]. Recently, it was proposed as an efficient tool to determine sampling and analysis strategies for NTD elimination surveys [53]. Soil-transmitted helminths (STH) are one of several NTDs for which MBG may become widely used [52,68–71]; the same framework can be applied to trachoma and, more broadly, to other NTDs. We advocate MBG as the new standard method to help NTD control programmes efficiently achieve their targets worldwide.

## Supporting information

S1 Text Supplementary Appendix. (DOCX)

## References

[1] Flaxman SR, Bourne RRA, Resnikoff S, Ackland P, Braithwaite T, Cicinelli M v., et al. Global causes of blindness and distance vision impairment 1990–2020: a systematic review and meta-analysis. Lancet Glob Health. 2017;5. doi: 10.1016/S2214-109X(17)30393-5 29032195

[2] Solomon AW, Burton MJ, Gower EW, Harding-Esch EM, Oldenburg CE, Taylor HR, et al. Trachoma. Nat Rev Dis Primers. 2022;8. doi: 10.1038/s41572-022-00359-5 35618795

[3] Miller K, Pakpour N, Yi E, Melese M, Alemayehu W, Bird M, et al. Pesky trachoma suspect finally caught. British Journal of Ophthalmology. 2004;88. doi: 10.1136/bjo.2003.038661 15148205 PMC1772198

[4] Versteeg B, Vasileva H, Houghton J, Last A, Abdurahman OS, Sarah V, et al. Viability PCR shows that non-ocular surfaces could contribute to transmission of chlamydia trachomatis infection in trachoma. PLoS Negl Trop Dis. 2020;14. doi: 10.1371/journal.pntd.0008449 32667914 PMC7384675

[5] Last A, Versteeg B, Abdurahman OS, Robinson A, Dumessa G, Aga MA, et al. Detecting extra-ocular chlamydia trachomatis in a trachoma-endemic community in ethiopia: Identifying potential routes of transmission. PLoS Negl Trop Dis. 2020;14. doi: 10.1371/journal.pntd.0008120 32130213 PMC7075638

[6] Habtamu E, Wondie T, Aweke S, Tadesse Z, Zerihun M, Zewdie Z, et al. Trachoma and Relative Poverty: A Case-Control Study. PLoS Negl Trop Dis. 2015;9. doi: 10.1371/journal.pntd.0004228 26600211 PMC4657919

[7] Wright HR, Turner A, Taylor HR. Trachoma and poverty: Unnecessary blindness further disadvantages the poorest people in the poorest countries. Clin Exp Optom. 2007;90. doi: 10.1111/j.1444-0938.2007.00218.x 17958564

[8] Emerson PM, Cairncross S, Bailey RL, Mabey DCW. Review of the evidence base for the “F” and “E” components of the SAFE strategy for trachoma control. Tropical Medicine and International Health. 2000;5. doi: 10.1046/j.1365-3156.2000.00603.x 10995092

[9] Garn J v., Boisson S, Willis R, Bakhtiari A, al-Khatib T, Amer K, et al. Sanitation and water supply coverage thresholds associated with active trachoma: Modeling cross-sectional data from 13 countries. PLoS Negl Trop Dis. 2018;12. doi: 10.1371/journal.pntd.0006110 29357365 PMC5800679

[10] Gambhir M, Basáñez MG, Turner F, Kumaresan J, Grassly NC. Trachoma: transmission, infection, and control. Lancet Infectious Diseases. 2007. doi: 10.1016/S1473-3099(07)70137-8 17521595

[11] Ramadhani AM, Derrick T, Holland MJ, Burton MJ. Blinding Trachoma: Systematic Review of Rates and Risk Factors for Progressive Disease. PLoS Neglected Tropical Diseases. 2016. doi: 10.1371/journal.pntd.0004859 27483002 PMC4970760

[12] Cromwell EA, Courtright P, King JD, Rotondo LA, Ngondi J, Emerson PM. The excess burden of trachomatous trichiasis in women: a systematic review and meta-analysis. Transactions of the Royal Society of Tropical Medicine and Hygiene. 2009. doi: 10.1016/j.trstmh.2009.03.012 19362326

[13] Kuper H, Solomon AW, Buchan J, Zondervan M, Foster A, Mabey D. A critical review of the SAFE strategy for the prevention of blinding trachoma. Lancet Infectious Diseases. 2003. doi: 10.1016/s1473-3099(03)00659-5 12781509

[14] Francis V, Turner V. Achieving community support for trachoma control: a guide for district health work (WHO/PBL/93.36). 1995.

[15] World Health Organization. Planning for the global elimination of trachoma (GET): report of a WHO consultation (WHO/PBL/97.60). Geneva, Switzerland; 1996.

[16] Solomon AW, Zondervan M, Kuper H, Buchan JC, Mabey DCW, Foster A. Trachoma control: a guide for program managers. Geneva, Switzerland; 2006.

[17] Solomon AW, Pavluck AL, Courtright P, Aboe A, Adamu L, Alemayehu W, et al. The Global Trachoma Mapping Project: Methodology of a 34-Country Population-Based Study. Ophthalmic Epidemiol. 2015;22. doi: 10.3109/09286586.2015.1037401 26158580 PMC4687001

[18] World Health Organization. WHO congratulates Benin and Mali for eliminating trachoma as a public health problem. 16 May 2023 [cited 23 May 2023]. Available from: https://www.who.int/news/item/16-05-2023-who-congratulates-benin-and-mali-for-eliminating-trachoma-as-a-public-health-problem.

[19] World Health Organization. Report of the 4th Global Scientific Meeting on Trachoma, Geneva, 27–29 November 2018. Geneva, Switzerland; 2019 Jun.

[20] Diggle PJ, Giorgi E. Model-based Geostatistics for Global Public Health. Model-based Geostatistics for Global Public Health. 2019. doi: 10.1201/9781315188492

[21] Amoah B, Fronterre C, Johnson O, Dejene M, Seife F, Negussu N, et al. Model-based geostatistics enables more precise estimates of neglected tropical-disease prevalence in elimination settings: Mapping trachoma prevalence in Ethiopia. Int J Epidemiol. 2022;51. doi: 10.1093/ije/dyab227 34791259 PMC9082807

[22] Brasil. Ministério da Saúde. Secretaria de Vigilância em Saúde. Departamento de Vigilância das Doenças Transmissíveis. Manual de vigilância do tracoma e sua eliminação como causa de cegueira. 2nd ed. 2014.

[23] Medina NH, Lopes MDF, Durkin SR, Cardoso MRA, Luna EA, Koizumi IK, et al. Survey of trachoma within school students in the state of Roraima, Brazil. Ophthalmology. 2011;118. doi: 10.1016/j.ophtha.2011.02.047 21684601

[24] Ferraz LCB, Schellini SA, Padovani CR, Medina NH, Dalben I. Prevalence of trachoma among school children in Bauru—São Paulo State, Brazil. Arq Bras Oftalmol. 2010;73. doi: 10.1590/S0004-27492010000500009 21225128

[25] Lopes MDFC Luna EJDA, Medina NH Cardoso MRA, Freitas HSDA Koizumi IK, et al. Prevalence of trachoma in Brazilian schoolchildren. Rev Saude Publica. 2013;47. doi: 10.1590/S0034-8910.2013047003428 24346557

[26] Luna EJ de A, Lopes M de FC, Medina NH, Favacho J, Cardoso MRA. Prevalence of Trachoma in Schoolchildren in Brazil. Ophthalmic Epidemiol. 2016;23. doi: 10.1080/09286586.2016.1244274 27824506

[27] Szwarcwald CL, Lopes M de FC, Borges de Souza Junior PR, Vaz Ferreira Gómez D, Luna EJ de A, da Silva de Almeida W, et al. Population Prevalence of Trachoma in Nine Rural Non-Indigenous Evaluation Units of Brazil. Ophthalmic Epidemiol. 2021. doi: 10.1080/09286586.2021.1941127 34711133 PMC10581672

[28] Hoechsmann A, Metcalfe N, Kanjaloti S, Godia H, Mtambo O, Chipeta T, et al. Reduction of trachoma in the absence of antibiotic treatment: Evidence from a population-based survey in Malawi. Ophthalmic Epidemiology. 2001. doi: 10.1076/opep.8.2.145.4169 11471084

[29] Tielsch JH, West KP, Katz J, Keyvan-Larijani E, Tizazu T, Schwab L, et al. The epidemiology of trachoma in southern Malawi. American Journal of Tropical Medicine and Hygiene. 1988;38. doi: 10.4269/ajtmh.1988.38.393 3354773

[30] Kalua K, Chirwa T, Kalilani L, Abbenyi S, Mukaka M, Bailey R. Prevalence and risk factors for trachoma in central and southern Malawi. PLoS One. 2010;5. doi: 10.1371/journal.pone.0009067 20140094 PMC2816719

[31] Kalua K, Chisambi A, Chinyanya D, Kamwendo Z, Masika M, Willis R, et al. Completion of Baseline Trachoma Mapping in Malawi: Results of Eight Population-Based Prevalence Surveys Conducted with the Global Trachoma Mapping Project. Ophthalmic Epidemiol. 2016;23. doi: 10.1080/09286586.2016.1230224 27726469 PMC5706967

[32] Kalua K, Phiri M, Kumwenda I, Masika M, Pavluck AL, Willis R, et al. Baseline Trachoma Mapping in Malawi with the Global Trachoma Mapping Project (GTMP). Ophthalmic Epidemiol. 2015;22. doi: 10.3109/09286586.2015.1035793 26158575 PMC4673584

[33] WHO validates Malawi for eliminating trachoma, first country in southern Africa. 2022 [cited 14 Nov 2022]. Available from: https://www.afro.who.int/news/who-validates-malawi-eliminating-trachoma-first-country-southern-africa.

[34] Cromwell EA, Amza A, Kadri B, Beidou N, King JD, Sankara D, et al. Trachoma prevalence in Niger: Results of 31 district-level surveys. Trans R Soc Trop Med Hyg. 2014;108. doi: 10.1093/trstmh/trt101 24281748

[35] World Health Organization. WHO Alliance for the Global Elimination of Trachoma: progress report on elimination of trachoma, 2021. Weekly epidemiological record. 2022;97: 353–364.

[36] World Health Organization. Design parameters for population-based trachoma prevalence surveys: strategic and technical advisory group for neglected tropical diseases, working group on monitoring and evaluation. 2018.

[37] World Health Organization. Technical consultation on trachoma surveillance: meeting report. September 11−12, 2014, Task Force for Global Health, Decatur, USA. 2015.

[38] Solomon AW, Willis R, Pavluck AL, Alemayehu W, Bakhtiari A, Bovill S, et al. Quality Assurance and Quality Control in the Global Trachoma Mapping Project. American Journal of Tropical Medicine and Hygiene. 2018;99. doi: 10.4269/ajtmh.18-0082 30039782 PMC6159583

[39] Thylefors B, Dawson CR, Jones BR, West SK, Taylor HR. A simple system for the assessment of trachoma and its complications. Bull World Health Organ. 1987;65. 3500800 PMC2491032

[40] Giorgi E, Fronterrè C, Macharia PM, Alegana VA, Snow RW, Diggle PJ. Model building and assessment of the impact of covariates for disease prevalence mapping in low-resource settings: To explain and to predict. Journal of the Royal Society Interface. 2021. doi: 10.1098/rsif.2021.0104 34062104 PMC8169216

[41] West SK, Munoz B, Turner VM, Mmbaga BBO, Taylor HR. The epidemiology of trachoma in central Tanzania. Int J Epidemiol. 1991;20. doi: 10.1093/ije/20.4.1088 1800408

[42] Courtright P, Sheppard J, Schachter J, Said ME, Dawson CR. Trachoma and blindness in the Nile Delta: Current patterns and projections for the future in the rural Egyptian population. British Journal of Ophthalmology. 1989;73. doi: 10.1136/bjo.73.7.536 2757994 PMC1041795

[43] Taylor HR, Burton MJ, Haddad D, West S, Wright H. Trachoma. The Lancet. 2014. doi: 10.1016/S0140-6736(13)62182-0 25043452

[44] Berry A, Hall J v. The Complexity of Interactions Between Female Sex Hormones and Chlamydia trachomatis Infections. Current Clinical Microbiology Reports. 2019. doi: 10.1007/s40588-019-00116-5 31890462 PMC6936955

[45] Brazilian Institute of Geography and Statistics (IBGE). Brazil Demographic Census 2010. Rio de Janeiro, Brazil; 2012.

[46] National Institute of Statistics of Niger. Statistics as a tool for decision making. [cited 16 Nov 2022]. Available from: https://www.stat-niger.org/?page_id=409.

[47] National Statistical Office of Malawi. 2018 Malawi Population and Housing Census. [cited 16 Nov 2022]. Available from: http://www.nsomalawi.mw/index.php?option=com_content&view=article&id=226&Itemid=6.

[48] WorldPop Hub. Population Density. [cited 3 Nov 2022]. Available from: https://hub.worldpop.org/geodata/listing?id=77.

[49] Giorgi E, Diggle PJ. PrevMap: An R package for prevalence mapping. J Stat Softw. 2017;78. doi: 10.18637/jss.v078.i08

[50] Global Administrative Areas (GADM). GADM data. [cited 26 May 2023]. Available: https://gadm.org/data.html

[51] Giorgi E, Osman AA, Hassan AH, Ali AA, Ibrahim F, Amran JGH, et al. Using non-exceedance probabilities of policy-relevant malaria prevalence thresholds to identify areas of low transmission in Somalia. Malar J. 2018;17. doi: 10.1186/s12936-018-2238-0 29463264 PMC5819647

[52] Johnson O, Fronterre C, Amoah B, Montresor A, Giorgi E, Midzi N, et al. Model-Based Geostatistical Methods Enable Efficient Design and Analysis of Prevalence Surveys for Soil-Transmitted Helminth Infection and Other Neglected Tropical Diseases. Clinical Infectious Diseases. 2021. doi: 10.1093/cid/ciab192 33905476 PMC8201574

[53] Fronterre C, Amoah B, Giorgi E, Stanton MC, DIggle PJ. Design and Analysis of Elimination Surveys for Neglected Tropical Diseases. Journal of Infectious Diseases. 2020;221. doi: 10.1093/infdis/jiz554 31930383 PMC7289555

[54] Burgert-Bruckern CR, Adams MW, Mingkwan P, Flueckiger R, Ngondi JM, Solomon AW, et al. Community-level trachoma ecological associations and the use of geospatial analysis methods: A systematic review. PLoS Negl Trop Dis. 2022;16. doi: 10.1371/journal.pntd.0010272 35395003 PMC9020723

[55] WorldPop. WorldPop gridded population estimate datasets and tools. How are they different and which should I use? [cited 17 Apr 2023]. Available from: https://www.worldpop.org/methods/populations/.

[56] Sanders AM, Abdalla Z, Elshafie BE, Elsanosi M, Nute AW, Aziz N, et al. Progress toward elimination of trachoma as a public health problem in seven localities in the republic of Sudan: Results from population-based surveys. American Journal of Tropical Medicine and Hygiene. 2019;101. doi: 10.4269/ajtmh.19-0530 31595874 PMC6896892

[57] Burton MJ, Hu VH, Massae P, Burr SE, Chevallier C, Afwamba IA, et al. What Is causing active trachoma? The role of nonchlamydial bacterial pathogens in a low prevalence setting. Invest Ophthalmol Vis Sci. 2011;52. doi: 10.1167/iovs.11-7326 21693601 PMC3176035

[58] Vasileva H, Butcher R, Pickering H, Sokana O, Jack K, Solomon AW, et al. Conjunctival transcriptome profiling of Solomon Islanders with active trachoma in the absence of Chlamydia trachomatis infection. Parasit Vectors. 2018;11. doi: 10.1186/s13071-018-2682-2 29467021 PMC5822555

[59] Lynch KD, Brian G, Ahwang T, Newie T, Newie V, Perrett C, et al. Discord between presence of follicular conjunctivitis and Chlamydia trachomatis infection in a single Torres Strait Island community: a cross-sectional survey. Aust N Z J Public Health. 2022;46. doi: 10.1111/1753-6405.13179 34978363

[60] Moraga P, Cano J, Baggaley RF, Gyapong JO, Njenga SM, Nikolay B, et al. Modelling the distribution and transmission intensity of lymphatic filariasis in sub-Saharan Africa prior to scaling up interventions: Integrated use of geostatistical and mathematical modelling. Parasit Vectors. 2015;8. doi: 10.1186/s13071-015-1166-x 26496983 PMC4620019

[61] Deshpande A, Miller-Petrie MK, Johnson KB, Abdoli A, Abrigo MRM, Adekanmbi V, et al. The global distribution of lymphatic filariasis, 2000–18: a geospatial analysis. Lancet Glob Health. 2020;8. doi: 10.1016/S2214-109X(20)30286-2 32827480 PMC7443698

[62] Hagan JE, Moraga P, Costa F, Capian N, Ribeiro GS, Wunder EA, et al. Spatiotemporal Determinants of Urban Leptospirosis Transmission: Four-Year Prospective Cohort Study of Slum Residents in Brazil. PLoS Negl Trop Dis. 2016;10. doi: 10.1371/journal.pntd.0004275 26771379 PMC4714915

[63] Amoah B, Giorgi E, Heyes DJ, van Burren S, Diggle PJ. Geostatistical modelling of the association between malaria and child growth in Africa. Int J Health Geogr. 2018;17. doi: 10.1186/s12942-018-0127-y 29482559 PMC5828493

[64] Macharia PM, Giorgi E, Noor AM, Waqo E, Kiptui R, Okiro EA, et al. Spatio-temporal analysis of Plasmodium falciparum prevalence to understand the past and chart the future of malaria control in Kenya. Malar J. 2018;17. doi: 10.1186/s12936-018-2489-9 30257697 PMC6158896

[65] Diggle PJ, Thomson MC, Christensen OF, Rowlingson B, Obsomer V, Gardon J, et al. Spatial modelling and the prediction of Loa loa risk: Decision making under uncertainty. Ann Trop Med Parasitol. 2007;101. doi: 10.1179/136485913X13789813917463 17716433

[66] Zouré HGM, Wanji S, Noma M, Amazigo UV, Diggle PJ, Tekle AH, et al. The geographic distribution of Loa loa in Africa: Results of large-scale implementation of the rapid assessment procedure for Loiasis (RAPLOA). PLoS Negl Trop Dis. 2011;5. doi: 10.1371/journal.pntd.0001210 21738809 PMC3125145

[67] Chipeta MG, Giorgi E, Mategula D, Macharia PM, Ligomba C, Munyenyembe A, et al. Geostatistical analysis of Malawi’s changing malaria transmission from 2010 to 2017. Wellcome Open Res. 2019;4. doi: 10.12688/wellcomeopenres.15193.2 31372502 PMC6662685

[68] Karagiannis-Voules DA, Biedermann P, Ekpo UF, Garba A, Langer E, Mathieu E, et al. Spatial and temporal distribution of soil-transmitted helminth infection in sub-Saharan Africa: A systematic review and geostatistical meta-analysis. Lancet Infect Dis. 2015;15. doi: 10.1016/S1473-3099(14)71004-7 25486852

[69] Chammartin F, Scholte RGC, Guimarães LH, Tanner M, Utzinger J, Vounatsou P. Soil-transmitted helminth infection in South America: A systematic review and geostatistical meta-analysis. Lancet Infect Dis. 2013;13. doi: 10.1016/S1473-3099(13)70071-9 23562238

[70] Pullan RL, Gething PW, Smith JL, Mwandawiro CS, Sturrock HJW, Gitonga CW, et al. Spatial modelling of soil-transmitted helminth infections in Kenya: A disease control planning tool. PLoS Negl Trop Dis. 2011;5. doi: 10.1371/journal.pntd.0000958 21347451 PMC3035671

[71] Pullan RL, Smith JL, Jasrasaria R, Brooker SJ. Global numbers of infection and disease burden of soil transmitted helminth infections in 2010. Parasit Vectors. 2014;7. doi: 10.1186/1756-3305-7-37 24447578 PMC3905661

