# Supplementary material for: Using model-based geostatistics for assessing the elimination of trachoma
Source: PLoS Negl Trop Dis. 2023 Jul 28;17(7):e0011476. doi: 10.1371/journal.pntd.0011476 (PMC10381061; doi:10.1371/journal.pntd.0011476)
Supplement: S1 Text — (DOCX) [file pntd.0011476.s001.docx]

**Supplementary Appendix**

This supplementary appendix provides methodological details for the geostatistical models fitted to trachomatous inflammation—follicular (TF) and trachomatous trichiasis (TT) prevalence. Based on these models, we also give technical details on how to predict prevalence and obtain the likelihood of having achieved the elimination target defined by the World Health Organization (WHO). We then compare the results obtained by our model-based geostatistics (MBG) and standard analysis methods.

1. **Geostatistical models for TF and TT**

The geostatistical Binomial models for TF and TT prevalence in Brazil, Malawi, and Niger take the following expressions.

TF model

$log\left\{ \frac{p_{j}\left( x_{i} \right)}{1-p_{j}\left( x_{i} \right)} \right\}= \alpha+\mathcal{f}\left( a_{ij} \right)+S\left( x_{i} \right)+Z_{i}$,

$\mathcal{f}\left( a_{ij} \right)=\beta_{1}a_{ij}+\beta_{2}\max\left( a_{ij}-3, 0 \right)$.

TT model

1. If interaction between age and gender is statistically significant

$$log\left\{ \frac{p_{j}\left( x_{i} \right)}{1-p_{j}\left( x_{i} \right)} \right\}= \alpha+\beta a_{ij}+\gamma d_{ij}\mathcal{+g}\left( a_{ij},d_{ij} \right)+S\left( x_{i} \right)+Z_{i}$$

$\mathcal{g}\left( a_{ij},d_{ij} \right)=\left\{ \begin{aligned} \delta_{\mathcal{F}}a_{ij} if female \\ \delta_{\mathcal{M}}a_{ij} if male \end{aligned} \right.$.

1. If interaction between age and gender is not statistically significant

$log\left\{ \frac{p_{j}\left( x_{i} \right)}{1-p_{j}\left( x_{i} \right)} \right\}= \alpha+\beta a_{ij}+\gamma d_{ij}+S\left( x_{i} \right)+Z_{i}$.

In the above expressions, $p_{ij}\left( x_{i} \right)$ represents the probability that an individual $j$ living in cluster $x_{i}$ has TF or TT. $\alpha$ and $\mathcal{d}$ indicate the age group and gender, respectively. $S\left( x \right)$ is a stationary and isotopic Gaussian process with mean zero, variance $\sigma^{2}$, and correlation function $\rho\left( u \right)=Corr\left( S\left( x \right),S\left( x^{'} \right) \right)=exp\left\{ -\left| x - x^{'} \right|/\phi\right\}$, where $\phi$ regulates how rapidly the spatial correlation decays with increasing distance between $x$ and $x^{'}$. $Z_{i}$ is independent and identically distributed Gaussian random variables with mean zero and variance $\tau^{2}$. The final models for each disease and country are shown in Table A. Note that for TT, Model I was not used in our analysis as the interaction between age and gender was not statistically significant in any country. We present it here as part of the model-building process, because the interaction might be an important predictor in other settings as described in the manuscript. We estimated the parameters by Monte Carlo maximum likelihood (MCML) using Prevmap (an R package) [1].

**Table A. Final form of the model in each disease and country**

| Model \ Country | Brazil | Malawi | Niger |
| --- | --- | --- | --- |
| TF | $\alpha+\beta a_{ij}+S\left( x_{i} \right)+Z_{i}$ | $\alpha+\beta_{1}a_{ij}+\beta_{2}\max\left( a_{ij}-3, 0 \right)+S\left( x_{i} \right)+Z_{i}$ | $\alpha+\beta_{1}a_{ij}+\beta_{2}\max\left( a_{ij}-3, 0 \right)+S\left( x_{i} \right)+Z_{i}$ |
| TT | $\alpha+\beta a_{ij}+\gamma d_{ij}+S\left( x_{i} \right)+Z_{i}$ | $\alpha+\beta a_{ij}+\gamma d_{ij}+S\left( x_{i} \right)+Z_{i}$ | $\alpha+\beta a_{ij}+\gamma d_{ij}+S\left( x_{i} \right)+Z_{i}$ |

1. **Prediction**

We predicted prevalence by first laying grid squares over evaluation units (EUs). Here we use the areas illustrated in Fig A as our EUs. The side of the squares was determined as the distance at which the spatial correlation is 90%. However, as the area of EUs was too large, we used the spatial correlation of 75% for both TF and TT in Brazil and 80% for TT in Niger.

We use a regular grid $x^{*}$ covering each analysed EU to approximate the prevalence $P_{k}$ for each class $k$ of age for TF and age and gender for TT, defined as

$$P_{k}=\left\{ P_{k}\left( x^{*} \right): x^{*}\in\mathrm{EUs} \right\}$$

We then standardised the prevalence according to age distributions for TF, and age and gender for TT, i.e.,

$$P\left( x^{*} \right)=\sum_{k} \mathcal{W}_{k}\left( x^{*} \right)P_{k}\left( x^{*} \right)$$

where $\mathcal{W}_{k}\left( x^{*} \right)$ is the proportion of the population in each class $k$ at location $x^{*}$. As population data we used EU-specific census data in Brazil (2010) [2] and national census data in Malawi (2018) and Niger (2012) [3,4].

Finally we computed the EU-wide prevalence accounting for population density i.e.,

$$P\left( EU \right)=\sum_{\mathcal{x*\in}EU} P\left( \mathcal{x*} \right)Pop\left( \mathcal{x*} \right)/\sum_{\mathcal{x*\in}EU} Pop\left( \mathcal{x*} \right)$$

where $Pop$ represents the population density according to WorldPop [5] (Fig B-D).

We obtained 10,000 predictive samples by MCML and then computed: (i) the point prediction of the EU-wide standardised average prevalence, using the mean of the predictive samples; (ii) the 95% level prediction intervals, using the 2.5^th^ and 97.5^th^ range of the samples; and (iii) the probability of elimination, computed as the proportion of the samples of $P\left( EU \right)$ that fall below the elimination threshold.


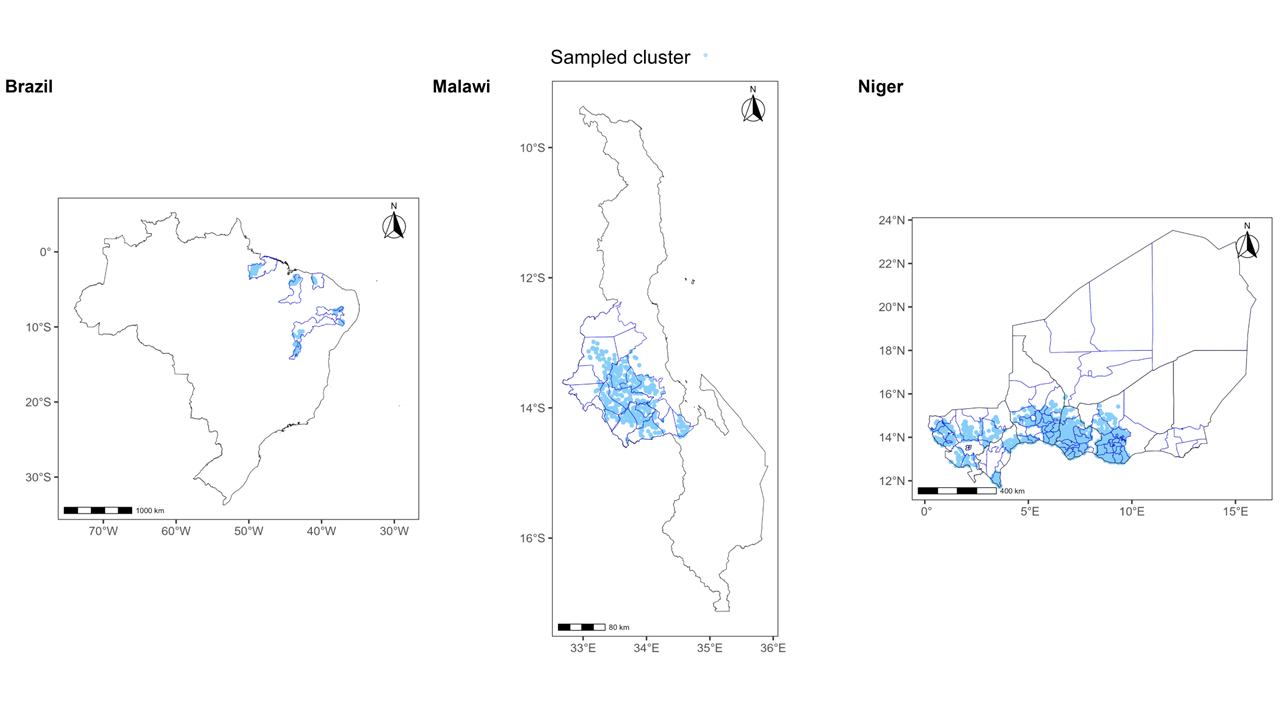


**Fig A. Surveyed locations in Brazil, Malawi, and Niger.** The blue points show the sampled locations, and the black and blue boundaries represent the boundaries of a country and prediction areas, respectively. The boundaries and names shown and the designations used on this map are based on Global Administrative Areas (GADM) [6] and do not imply the expression of any opinion whatsoever on the part of the authors, or the institutions with which they are affiliated, concerning the legal status of any country, territory, city or area or of its authorities, or concerning the delimitation of its frontiers or boundaries.

**
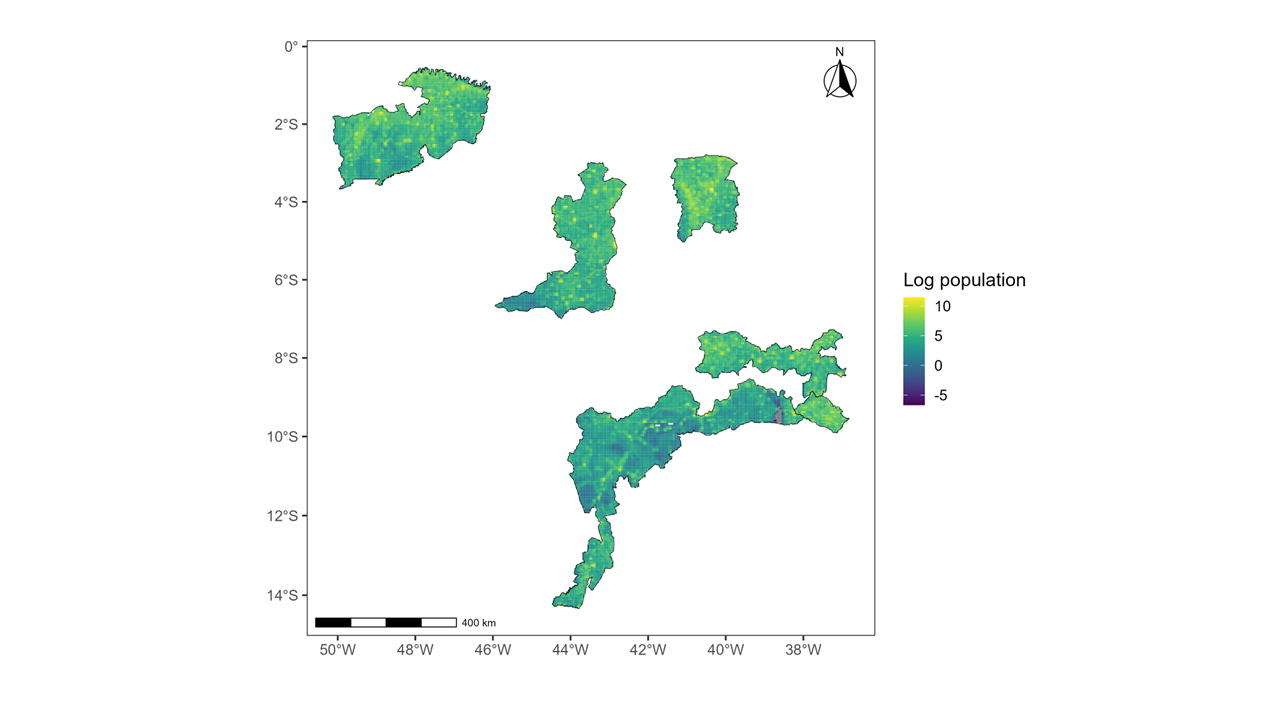
**

**Fig B. The log population in evaluation units (EUs) in Brazil according to WorldPop data** [5] **on a 5km grid.** The grey area shows where the population is 0. The boundaries shown and the designations used on this map are based on Global Administrative Areas (GADM) [6] and do not imply the expression of any opinion whatsoever on the part of the authors, or the institutions with which they are affiliated, concerning the legal status of any country, territory, city or area or of its authorities, or concerning the delimitation of its frontiers or boundaries.


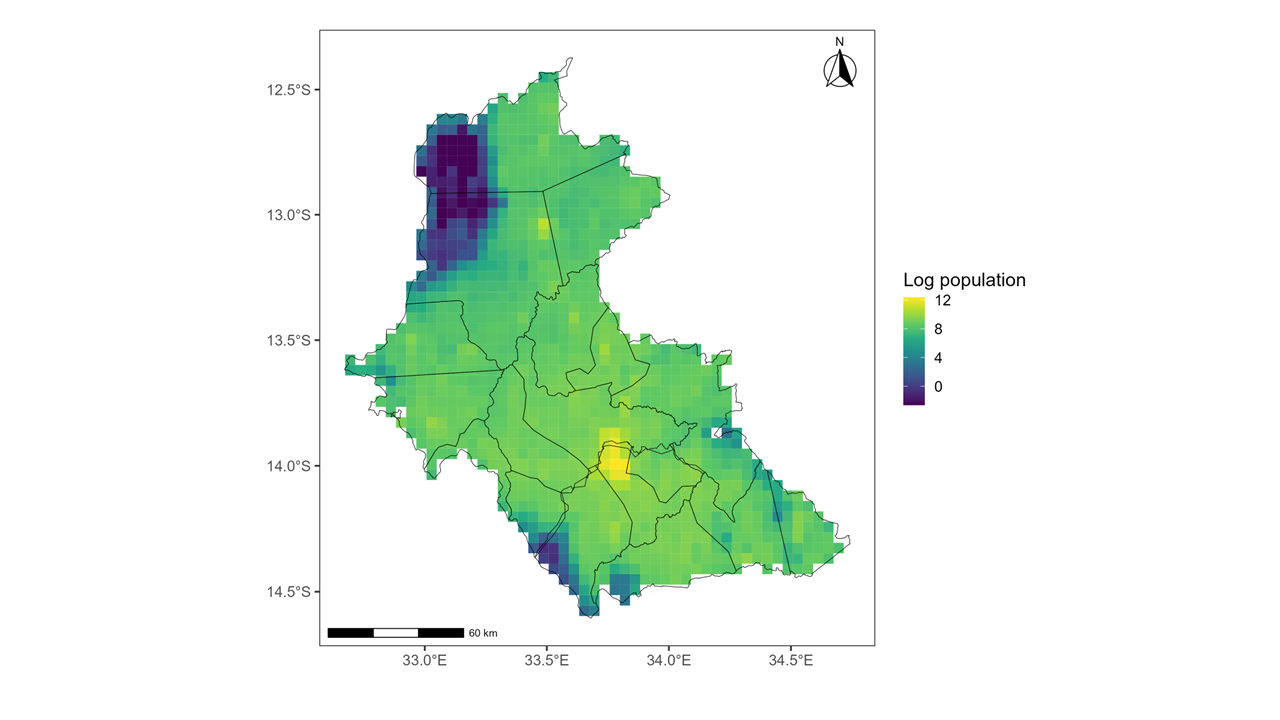


**Fig C. The log population in evaluation units (EUs) in Malawi according to WorldPop data** [5] **on a 5km grid.** The boundaries shown and the designations used on this map are based on Global Administrative Areas (GADM) [6] and do not imply the expression of any opinion whatsoever on the part of the authors, or the institutions with which they are affiliated, concerning the legal status of any country, territory, city or area or of its authorities, or concerning the delimitation of its frontiers or boundaries.


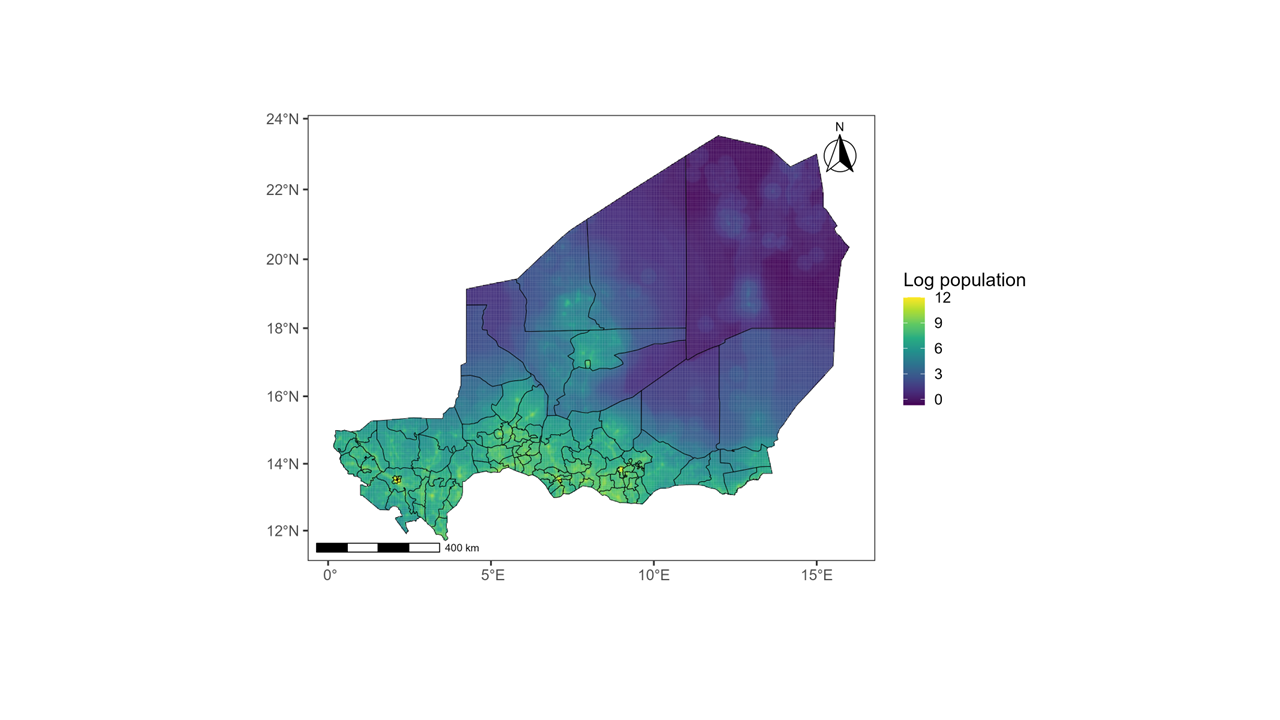


**Fig D. The log population in evaluation units (EUs) in Niger according to WorldPop data** [5] **on a 5km grid.** The boundaries shown and the designations used on this map are based on Global Administrative Areas (GADM) [6] and do not imply the expression of any opinion whatsoever on the part of the authors, or the institutions with which they are affiliated, concerning the legal status of any country, territory, city or area or of its authorities, or concerning the delimitation of its frontiers or boundaries.

1. **Comparison with conventional approach**

Table B and C compare our predicted TF and TT prevalence in Brazil with that computed according to standard analysis methods [7]. The results were consistent with each other, as 95% predictive intervals overlap. The only discrepancy was found in TF prevalence in Vale São do Francisco da Bahia, where there was only one TF case of 614 examined.

**Table B. Comparison of predicted TF prevalence based on MBG and standard methods**

|  | Predicted TF prevalence (95% predictive interval) | |
| --- | --- | --- |
| EU | MBG | Standard |
| Nordeste Paraense | 0.94 (0.46–2.18) | 0.96 (0.29–1.88) |
| Leste Maranhense | 0.91 (0.333–2.71) | 0.13 (0.00–0.39) |
| Noroeste Cearense | 0.91 (0.39–2.16) | 0.58 (0.00–1.50) |
| Sertão Pernambucano | 0.91 (0.37–2.41) | 0.20 (0.00–0.51) |
| Sertão Alagoano | 0.72 (0.21–2.21) | 0.00 (0.00–0.00) |
| Vale São do Francisco da Bahia | 0.85 (0.34–2.35) | 0.08 (0.00–0.24) |

**Table C. Comparison of predicted TT prevalence based on MBG and standard methods**

|  | Predicted TT prevalence (95% predictive interval) | |
| --- | --- | --- |
| EU | MBG | Standard |
| Nordeste Paraense | 0.09 (0.04–0.24) | 0.00 (0.00–0.00) |
| Leste Maranhense | 0.11 (0.04–0.32) | 0.00 (0.00–0.00) |
| Noroeste Cearense | 0.18 (0.06–0.44) | 0.22 (0.06–0.44) |
| Sertão Pernambucano | 0.14 (0.05–0.41) | 0.05 (0.00–0.12) |
| Sertão Alagoano | 0.09 (0.02–0.31) | 0.00 (0.00–0.00) |
| Vale São do Francisco da Bahia | 0.16 (0.05–0.56) | 0.05 (0.00–0.13) |

**Reference**

1. Giorgi E, Diggle PJ. PrevMap: An R package for prevalence mapping. J Stat Softw. 2017;78. doi:10.18637/jss.v078.i08

2. Ministry of Health B. DATASUS. [cited 16 Nov 2022]. Available: https://datasus.saude.gov.br/

3. National Institute of Statistics of Niger. Statistics as a tool for decision making. [cited 16 Nov 2022]. Available: https://www.stat-niger.org/?page_id=409

4. National Statistical Office of Malawi. 2018 Malawi Population and Housing Census. [cited 16 Nov 2022]. Available: http://www.nsomalawi.mw/index.php?option=com_content&view=article&id=226&Itemid=6

5. WorldPop Hub. Population Density. [cited 3 Nov 2022]. Available: https://hub.worldpop.org/geodata/listing?id=77

6. Global Administrative Areas (GADM). GADM data. [cited 26 May 2023]. Available: https://gadm.org/data.html

7. Szwarcwald CL, Lopes M de FC, Borges de Souza Junior PR, Vaz Ferreira Gómez D, Luna EJ de A, da Silva de Almeida W, et al. Population Prevalence of Trachoma in Nine Rural Non-Indigenous Evaluation Units of Brazil. Ophthalmic Epidemiol. 2021. doi:10.1080/09286586.2021.1941127
